# Dataset for Automatic Region-based Coronary Artery Disease Diagnostics Using X-Ray Angiography Images

**DOI:** 10.1038/s41597-023-02871-z

**Published:** 2024-01-03

**Authors:** Maxim Popov, Akmaral Amanturdieva, Nuren Zhaksylyk, Alsabir Alkanov, Adilbek Saniyazbekov, Temirgali Aimyshev, Eldar Ismailov, Ablay Bulegenov, Arystan Kuzhukeyev, Aizhan Kulanbayeva, Almat Kalzhanov, Nurzhan Temenov, Alexey Kolesnikov, Orazbek Sakhov, Siamac Fazli

**Affiliations:** 1 Mohamed Bin Zayed University of Artificial Intelligence, Department of Computer Vision, Abu Dhabi, United Arab Emirates; 2 Research Institute of Cardiology and Internal Diseases, Almaty, 050000 Kazakhstan; 3 CMC Technologies, Astana, 010000 Kazakhstan; 4 Almaty City Cardiological Center, Almaty, 050000 Kazakhstan; 5 Nazarbayev University, School of Engineering and Digital Sciences, Department of Computer Science, Astana, 010000 Kazakhstan

**Keywords:** Ischaemia, Radiography

## Abstract

X-ray coronary angiography is the most common tool for the diagnosis and treatment of coronary artery disease. It involves the injection of contrast agents into coronary vessels using a catheter to highlight the coronary vessel structure. Typically, multiple 2D X-ray projections are recorded from different angles to improve visualization. Recent advances in the development of deep-learning-based tools promise significant improvement in diagnosing and treating coronary artery disease. However, the limited public availability of annotated X-ray coronary angiography image datasets presents a challenge for objective assessment and comparison of existing tools and the development of novel methods. To address this challenge, we introduce a novel ARCADE dataset with 2 objectives: coronary vessel classification and stenosis detection. Each objective contains 1500 expert-labeled X-ray coronary angiography images representing: i) coronary artery segments; and ii) the locations of stenotic plaques. These datasets will serve as a benchmark for developing new methods and assessing existing approaches for the automated diagnosis and risk assessment of coronary artery disease.

## Background & Summary

Coronary artery disease (CAD) is a leading cause of death worldwide^1^. CAD contributes to about 18 million deaths annually, which comprises approximately one-third of worldwide mortality causes^2^. It is caused by the buildup of plaque in the coronary arteries, which can narrow or block the arteries and reduce blood flow to the heart^3^. CAD elicits a spectrum of severe clinical manifestations including angina pectoris, myocardial infarction, and abrupt cardiac death.

X-ray Coronary Angiography (XCA), or Invasive Coronary Angiography (ICA), is a commonly used imaging technique for identifying CAD. During XCA, a catheter is inserted into a blood vessel, and a contrast agent is injected into the coronary arteries^4^. The agent improves the visibility of the arteries on X-ray images, which doctors utilize to identify narrowing or blockages. Despite being a standard methodology, XCA images impose many challenges such as non-even illumination, low contrast, presence of other body tissues or a catheter which may lead to misdiagnosis^4^.

While existing literature supports the use of non-invasive Computed Tomography Angiography (CTA)-based methods, such as CTA-based Functional Flow Reserve (FFRCT) analysis, as potential replacements for XCA in the detection of intermediate CAD^5,6^, the documentation and analysis of severe and obstructive CAD using these methods remains unreliable. This unreliability is attributed to the significantly lower resolution, increased impact of visual artifacts, and the natural motion of vessels in CTA^7,8^. As a result, these methods only allow for the assessment of CAD’s presence but not its severity. Hence, most of the modern research in CAD diagnostics relies on XCA as the main quantitative method and it is often referred to as a golden standard in various sources^9,10^.

Quantitative coronary analysis (QCA) is a technique employed to assess the severity of CAD based on the location of lesions. QCA can be used to determine the risk of a heart attack or sudden cardiac death. It is a challenging and time-consuming task. However, recent advancements in deep learning have shown considerable progress in medical image processing and analysis, outperforming traditional methods and opening new possibilities for automating QCA. The decisive factor for developing automated diagnostic techniques is the availability of high-quality annotated data. To date, only a few curated datasets have been published covering semantic segmentation of coronary arteries. Moreover, many studies only use private datasets, hindering the ability to assess and compare existing and novel algorithms effectively.

To alleviate the lack of annotated medical data for CAD diagnostics, we provide a benchmark dataset for developing and evaluating automated algorithms for CAD diagnosis, utilizing XCA images. This dataset is published with the Automatic Region-based Coronary Artery Disease Diagnostics using X-ray angiography images (ARCADE)^11^ challenge, hosted under the 26th International Conference on Medical Image Computing and Computer-Assisted Intervention (MICCAI).

The ARCADE dataset consists of two instance segmentation tasks. The first task includes 1500 coronary vessel tree images and their respective annotations, depicting the division of a heart into 25 different regions based on the SYNTAX Score methodology^12^. The second task includes a different set of 1500 images with annotated regions containing atherosclerotic plaques. This dataset was carefully annotated by medical experts, enabling scientists to actively contribute towards the advancement of an automated risk assessment system for patients with CAD. To our knowledge, this dataset constitutes the largest expert-labeled and openly available collection of XCA images.

## Related Work

The following section provides an overview of several state-of-the-art frameworks and methodologies employed in the automated diagnosis of CAD using X-ray angiography. Each approach offers distinct contributions to the field, addressing challenges associated with coronary segmentation, catheter detection, and functional assessment.

Zhang *et al*. introduced a progressive perception learning (PPL) framework for main coronary segmentation in X-ray angiography^13^. This approach addresses challenges faced by traditional and existing deep learning methods by employing context, interference, and boundary perception modules. The context perception module focuses on the main coronary vessel by capturing semantic dependence among different coronary segments. The interference perception module purifies feature maps by enhancing the foreground vessel and suppressing background artifacts. The boundary perception module highlights boundary details through boundary feature extraction. This approach was tested on a dataset of 1086 subjects, achieving a Dice score exceeding 95% and outperforming thirteen state-of-the-art methods.

Fazlali *et al*. proposed a framework for coronary artery segmentation and catheter detection in x-ray angiography images, leveraging superpixels and vesselness probability measures^4^. The method employs multiple superpixel scales and a majority voting approach for initial segmentation, followed by orthogonal line refinement for vessel region delineation. Catheter detection and tracking utilize ridge detection and polynomial fitting. Image ridges are employed for coronary artery centerline extraction. The method is evaluated on 2 datasets, DS1 and DS2, comprising 164 and 298 images, respectively. The results demonstrate superior segmentation performance and a low false positive rate, all accomplished in a shorter time than the previously proposed graph-cut-based algorithm. The benefit of this method is its robustness and there is no need for labels for vessel segmentation.

Zhang *et al*.^14^ emphasized the significance of IoT-based smart health in diagnosing cardiovascular disease while highlighting the limitations of traditional hemodynamic parameter assessment methods. To address these challenges, the authors introduced a physics-guided deep learning network that integrates knowledge about blood flow into the loss function for accurate and physiologically consistent functional assessment. Their approach incorporates an attentive network to identify crucial coronary artery anatomy features and segments. Extensive experiments on 20000 synthetic and 140 clinical subject data, including blood pressure and velocity inside vessels, showcase the method’s accuracy and interpretability in stenosis categorization, promoting the broader adoption of IoT and deep learning in the realm of smart health.

The most relevant work was conducted by Du *et al*. This study utilized a dataset comprising 20,612 coronary angiograms, with 13,373 angiograms labeled for coronary artery segments and 7,239 labeled for special lesion morphology^15^. A deep learning architecture was trained and optimized for automatic analysis of coronary angiography, featuring two networks: one for recognizing 20 coronary artery segments and another for detecting various lesion morphologies. Their segment prediction network achieved a high recognition accuracy of 98.4% and sensitivity of 85.2%. For lesion morphology detection, F1 scores ranged from 0.802 to 0.854, with the entire recognition process taking just two seconds. This deep learning approach provides a comprehensive coronary diagnostic map, aiding cardiologists in identifying and diagnosing lesion severity and morphology during interventions.

However, one common disadvantage of these methods is the unavailability of the data they were trained on. This makes it impossible to reproduce, validate, and build on top of them. With this dataset, we are trying to reduce this gap and provide a solid basis for future research in the area of automated diagnosis of CAD.

The ARCADE dataset, introduced in this paper, stands out as a substantial contribution to the field of XCA analysis, offering several advantages over existing datasets. With 1,500 images represented, the ARCADE dataset is significantly larger and hence more diverse than many existing datasets. This diversity better reflects the real-world patient population and variations in XCA images. Similar to existing datasets, the ARCADE dataset provides static images with structural annotations. However, it goes beyond by offering the potential for analyzing the complexity of CAD based on the locations of individual vessels. In addition, our dataset includes annotations related to stenotic plaques, enabling the development of risk assessment models for patients with CAD. Published alongside the MICCAI challenge, the ARCADE dataset invites the research community to actively participate in advancing CAD diagnostics and XCA analysis. It provides a solid baseline for future research in this field.

In the landscape of open datasets for XCA analysis, the ARCADE dataset distinguishes itself by its size, diversity, and more complex objective. Researchers in the field can leverage this dataset to develop more robust and accurate automated XCA analysis methods, ultimately improving CAD diagnostics and patient care.

## Methods

### Ethical statement

All samples were collected retrospectively from the X-ray and vascular surgery archive of the Research Institute of Cardiology and Internal Diseases with approval to publish under an open license from the Local Ethical Commission of NJSC “Asfendiyarov Kazakh National Medical University” (LECAKNMU #1121). The ethical committee waived the consent due to the retrospective nature of the study.

### Patient cohort

The study cohort consists of patients with suspected CAD, whose clinical data is available at the Research Institute of Cardiology and Internal Diseases, Almaty, Kazakhstan. The total number of patients is 1500 with a Mean age of 45.8, Median age of 60.0, 57% men (youngest 21, oldest 85), and 43% women (youngest 19, oldest 90).

### Imaging

XCA images were acquired using two different angiographs: the Philips Azurion 3 (Philips, Amsterdam, Netherlands) and the Siemens Artis Zee (Siemens Medical Solutions, Erlangen, Germany). The dimensions of the collected XCA images are 512 × 512 pixels. Omnipaque and Visipaque contrast media were used for imaging.

The XCA images were acquired using a standardized protocol. All patients were positioned in a supine orientation on an angiography table. The patient’s heart rate was controlled at around 70–85 bpm by administration of beta blockers. The femoral artery was accessed with a sheath and a catheter was advanced into the coronary arteries. A contrast media was injected into the bloodstream and 4 views for the left coronary artery (LCA) and 2 views for the right coronary artery (RCA) were obtained including Left Anterior Oblique (LAO) and Right Anterior Oblique (RAO) caudal views, Postero-Anterior (PA) and RAO cranial views for LCA, and LAO and RAO cranial views for RCA. Subsequently, the XCA images were stored in the Digital Imaging and Communications in Medicine (DICOM) format and transferred to a computer for further analysis. XCA images typically include metadata such as the patient’s name, date of screening, image modality, and angiographic device. Due to privacy concerns, the metadata was removed from the DICOM files except for the sequence of frames.

### Frame selection

The XCA videos used to construct this dataset were recorded from six viewing angles, with approximately 60 to 120 frames per video. This resulted in a range of 300 to 800 images per patient. Based on the aforementioned criteria, 0 to 2 images were selected per viewing angle for subsequent annotation. Therefore, this dataset may contain 0 to 12 frames from a single patient. The frame selection process emphasized several key criteria. First, images were chosen based on optimal contrast filling within coronary arteries to ensure effective visualization. Second, preference was given to images with minimal blurriness or motion artifacts to maintain high image quality. Third, frames capturing the target lesion or lesions, particularly stenotic plaques, were prioritized for inclusion. Lastly, the dataset aimed to encompass clinically relevant cases, including those with three-vessel diseases, providing a comprehensive representation of coronary artery disease scenarios for research and development. Also, to improve the variability of data, left anterior descending (LAD), left circumflex artery (LCX), and right coronary artery (RCA) views were represented in equal proportions in this dataset, providing a wide range of clinical cases for analysis.

In total, 3,000 frames were collected. 1,500 frames were selected for artery classification and 1,500 frames for stenosis detection tasks. These frames were then split into training, public validation, and private testing sets, with 1000, 200, and 300 samples in each partition.

### Annotation

Computer Vision Annotation Tool (CVAT) software (10.5281/zenodo.3497106) was used for annotating XCA images. It is a web-based tool that allows cardiologists to create and edit annotations and assign labels to them. To the best of our knowledge, CVAT software provides state-of-the-art capabilities for pixel-level annotations. By performing multi-step verification with the help of experienced cardiologists we ensure precise annotation. Also, even approximate locations of vessel regions are enough to guide a cardiologist in diagnosing CAD.

The SYNTAX methodology^12^ was used as a basis for coronary vessel classification. It is a scoring system that quantifies the severity of coronary artery disease based on the location and type of lesions.

The specialists used a standardized annotation protocol to ensure the consistency and accuracy of the annotations. According to this protocol, there are three major coronary arteries: the RCA, the LCX, and the LAD. LCX and LAD share a common origin, the left main coronary artery (LMCA). In addition, each of the three main arteries is commonly divided into three sub-segments, namely RCA proximal, RCA mid, RCA distal; proximal circumflex, distal circumflex, and posterior descending (in case of left coronary dominance); LAD proximal, LAD mid, and LAD distal. These sub-segments also have their own sets of side branches, such as obtuse margins in LCX, and diagonal segments in LAD, which are also defined in the protocol. Each of the sub-segments is assigned a unique alphanumeric identifier, ranging from 1 to 16*c*, which is used for shorter notation instead of full class names in the dataset. A higher numeric value indicates a deeper position and/or lower importance in the coronary tree, with subsequent segments always having a higher number than the segments they originate from. For smaller branches, such as obtuse margins, posterolateral, and diagonals, they are denoted with alphabet characters *a* to *c* to signify their relative positions from the origin, with *a* being the closest and *c* being the farthest ones. Full correspondence between class names and their identifier is provided in the SYNTAX methodology^12^.

In addition, the locations of stenotic plaques (stenosis) were annotated. According to the definition given by SYNTAX Score, stenosis is defined as a coronary lesion with 50% and higher narrowing in vessels with a thickness of 1.5 *mm* or more. The stenotic plaques were evaluated using built-in QCA software in angiography systems and visually by cardiologists. Every signal included in the dataset was originally annotated by one expert. Subsequently, the whole image collection was cross-validated by two doctors with the highest expertise in the team. The final annotation was established based on the consensus between the two cross-validating doctors to ensure high-quality annotations.

## Data Records

The dataset is available at the ARCADE^11^ Zenodo repository under 10.5281/zenodo.10390295. The dataset structure is as follows: top-level directories “syntax” and “stenosis” contain files for the two dataset objectives, namely: i) vessel branch classification according to the SYNTAX methodology; and ii) stenosis detection. Inside both directories, there are 3 subsets of the dataset, such as “train”, “val”, and “test”. Inside each of those folders, there are 2 lower-level directories - “images”, and “annotations”. Inside the “images” folder there are images in “.png” format, extracted from DICOM recordings. The “annotations” folders contain single “.JSON” files, which are named in correspondence to the objective, i.e. “train.JSON”, “val.JSON”, and “test.JSON”.

The structure of “.JSON” contains three top-level fields: “images”, “categories”, and “annotations”. The “images” field contains the unique “id” of the image in the dataset, its “width” and “height” in pixels, and the “file_name” sub-field, which contains specific information about the image. The “categories” field contains a unique “id” from 1 to 26, and a “name”, relating it to the SYNTAX descriptions. The “annotations” field contains a unique “id” of the annotation, “image_id” value, relating it to the specific image from the “images” field, and a “category_id” relating it to the specific category from the “categories” field. The “segmentation” sub-field contains coordinates of mask edge points in “XYXY” format. Bounding box coordinates are given in the “bbox” field in the “XYWH” format, where the first 2 values represent the *x* and *y* coordinates of the left-most and top-most points in the segmentation mask. The height and width of the bounding box are determined by the difference between the right-most and bottom-most points and the first two values. Finally, the “area” field provides the total area of the bounding box, calculated as the area of a rectangle.

## Technical Validation

### Annotation assessment

To assess the consistency of labeling across interventional cardiologists, we designed a benchmark task consisting of 10 sample images and requested all six doctors to label them. The task involved annotating the majority of important vessels on the XCA images, including diagonal arteries and obtuse margins. These vessels, which happen to be close to the limit of the XCA resolution, were carefully annotated by the team. Based on their combined annotations, we manually derived a meta-label. The similarity of individual labeling and the meta-label was computed with the help of the Dice Similarity Coefficient (DSC):

1 $${\rm{DSC}}=\frac{2\cdot ({\rm{Precision}}\cdot {\rm{Recall}})}{{\rm{Precision}}+{\rm{Recall}}},\quad {\rm{Precision}}=\frac{TP}{TP+FP},\quad {\rm{Recall}}=\frac{TP}{TP+FN}$$

where TP stands for true positives, FP for false positives, and FN for false negatives.

**Fig. 1:**
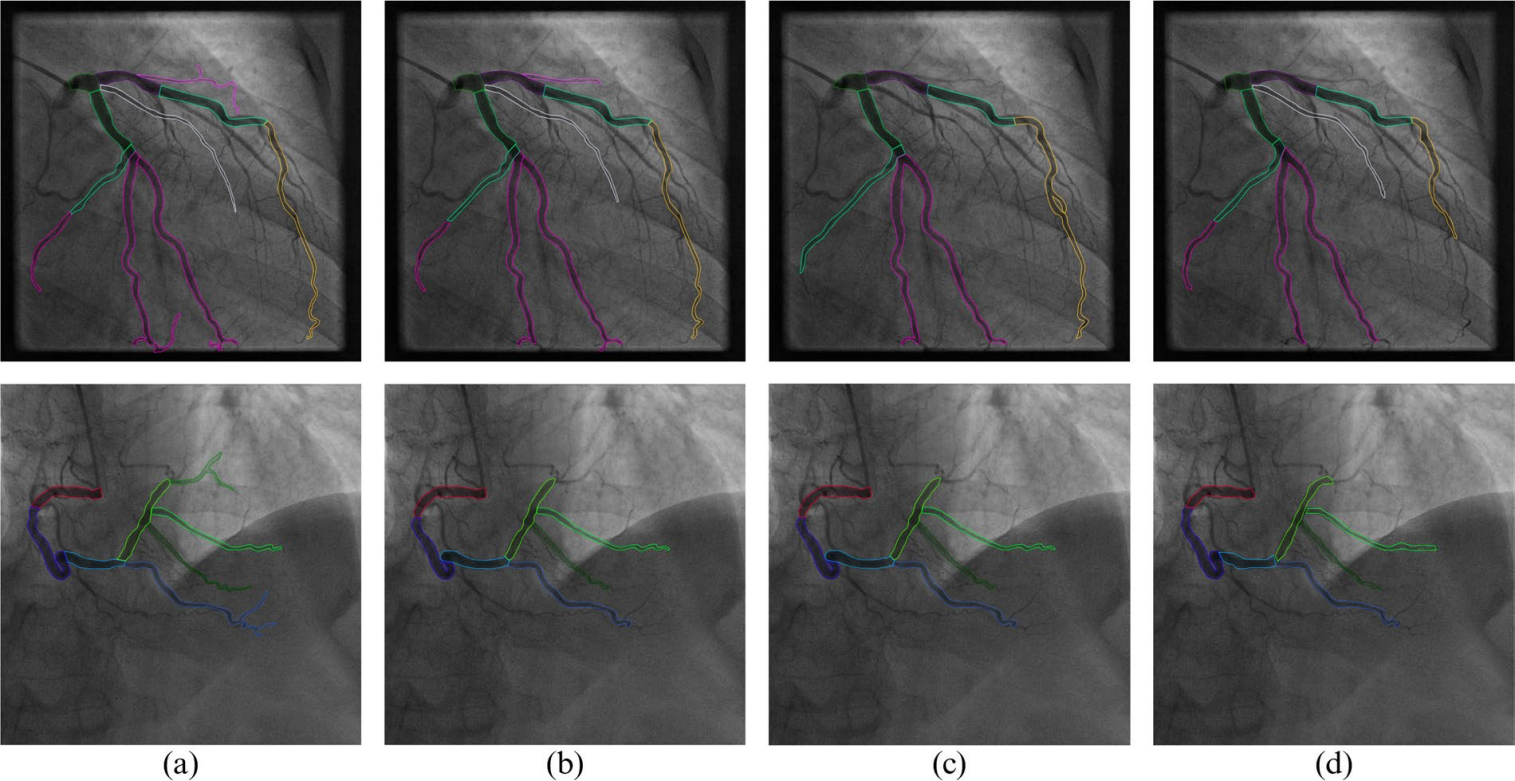
Image annotations by different annotators. Column (**a**) - ground truth annotation, which includes most of the vessels described in the SYNTAX; (**b**–**d**) - annotations by individual doctors.

The results can be seen in Table 1, where a DSC of 1 indicates maximum similarity. Examples of disagreement in the data are presented in Fig. 1. Our results indicate a high level of agreement among the annotators, where most disagreements can be attributed to minor differences in class borders and the inclusion/exclusion of smaller vessels in the annotation, both do not affect the clinical value of the data significantly.

**Table 1:** Dice Scores estimating the similarity of labeling across annotators.

Ann.	1	2	3	4	5	6	7	8	9	10
A1	0.89	0.75	0.74	0.8	0.84	0.7	0.81	0.77	0.79	0.64
A2	0.81	0.7	0.89	0.83	0.92	0.91	0.76	0.67	0.89	0.62
A3	0.87	0.79	0.77	0.82	0.92	0.75	0.62	0.72	0.77	0.74
A4	0.9	0.93	0.94	0.97	0.96	0.88	0.96	0.95	0.91	0.95
A5	0.77	0.7	0.8	0.83	0.91	0.82	0.84	0.8	0.81	0.8
A6	0.79	0.81	0.8	0.86	0.84	0.7	0.76	0.75	0.77	0.62
Mean	0.84	0.78	0.82	0.85	0.9	0.79	0.79	0.78	0.83	0.73
Std Dev	0.0464	0.0860	0.0795	0.0681	0.0681	0.0901	0.1169	0.1124	0.0679	0.1421

### Deep learning model training

To improve the contrast between the background and the vessels, we sequentially applied the white top-hat transformation, with a kernel of size 50 × 50 containing ‘1’ s, on the negative of the image and subtracted the result from the original image. Subsequently, we clipped the result from 0 to 255, and finally applied the Contrast Limited Adaptive Histogram^16^ with a grid size of 8 × 8 and a clip limit of 2. The white top-hat transformation is a morphological operation that can enhance the sharpness of vessel edges. The Contrast Limited Adaptive Histogram algorithm is a histogram equalization algorithm that can be used to improve the contrast between the vessels and the background.

**Fig. 2:**
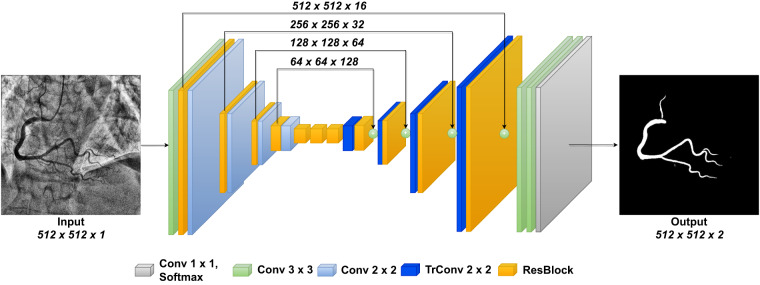
U-Net Architecture.

To test the suitability of this dataset for semantic vessel segmentation, we implemented a Residual U-Net^17^. It is a convolutional neural network that follows an encoder-decoder structure, with the encoder responsible for extracting features from the image, and the decoder responsible for reconstructing the binary segmentation mask from the extracted features. This model utilizes residual blocks and skip connections that link the encoder to the decoder, allowing the model to better preserve the vessel structure. A schematic representation of the architecture can be found in Fig. 2.

**Fig. 3:**
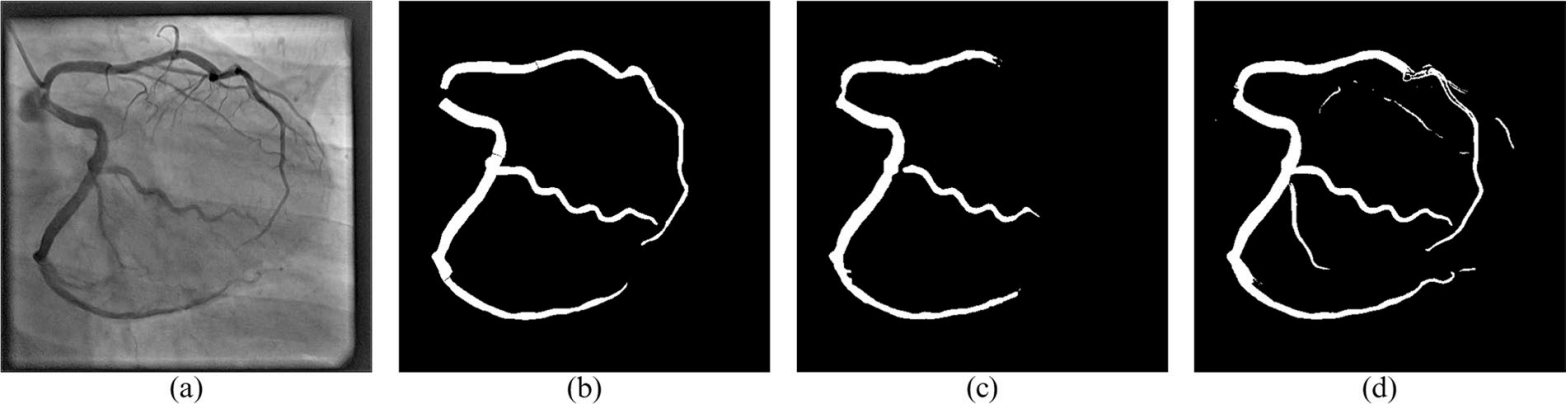
Semantic segmentation of vessels using the ARCADE dataset: (**a**) original XCA image, (**b**) ground truth binary mask, (**c**) U-Net binary segmentation using standard weights, (**d**) Segmentation using our weighted-edge approach.

This model was trained with XCA images as an input (Fig. 3a) and corresponding binary masks as targets (Fig. 3b). However, the vessel-background ratio on XCA images is meager. Additionally, only approximately 60–80% of the total area of the vasculature is annotated in the masks, since cardiologists only labeled the most important vessels on each image. This leads to a potential problem, since U-Net may misinterpret unlabeled vessels as background, thus diminishing the segmentation performance, especially for smaller vessel branches. To partially overcome this limitation, we introduce a custom loss function, where pixels of target images that correspond to vessel borders are given higher importance.

In particular, a gradient-based edge detection algorithm was applied to the target image, and resulting values of horizontal and vertical gradients were squared and summed, forming an edge mask. The gradient is then calculated again, resulting in an image, highlighting the vessel edges. Edge pixel positions were then used to create a pixel-weighted loss function, thus penalizing the model less for including additional vessel-like structures, or the false positives, in the output and penalizing the model more for incorrect identification of vessel edges or the false negatives. As a result, the model started to pay attention to vessels that were not included in the original binary masks, as shown in Fig. 3d. To test the performance of this model, we have fine-tuned it on a holdout set of 100 images from the DCA1^18^ and tested its performance on the remaining 34 images. The mean Dice score of this model trained on our dataset and fine-tuned on DCA1 achieved 0.75, and the mean Recall showed 0.84. For comparison, the model trained only on a tuning subset of DCA1 reached a Dice score of 0.59 and a Recall of 0.69, which shows the usefulness of the ARCADE dataset for binary segmentation tasks.

To provide a baseline model for multi-class segmentation, we trained YOLOv8 (https://github.com/ultralytics/ultralytics), a state-of-the-art object detection algorithm, that can be used to detect coronary arteries and stenosis in XCA images. The proposed methodology consists of two stages: object detection and segmentation. In the first stage, YOLOv8 identifies the locations of vessels and stenoses in the image and assigns corresponding labels. In the second stage, the segmentation heads are employed to predict pixel-wise masks for the coronary arteries.

**Fig. 4:**
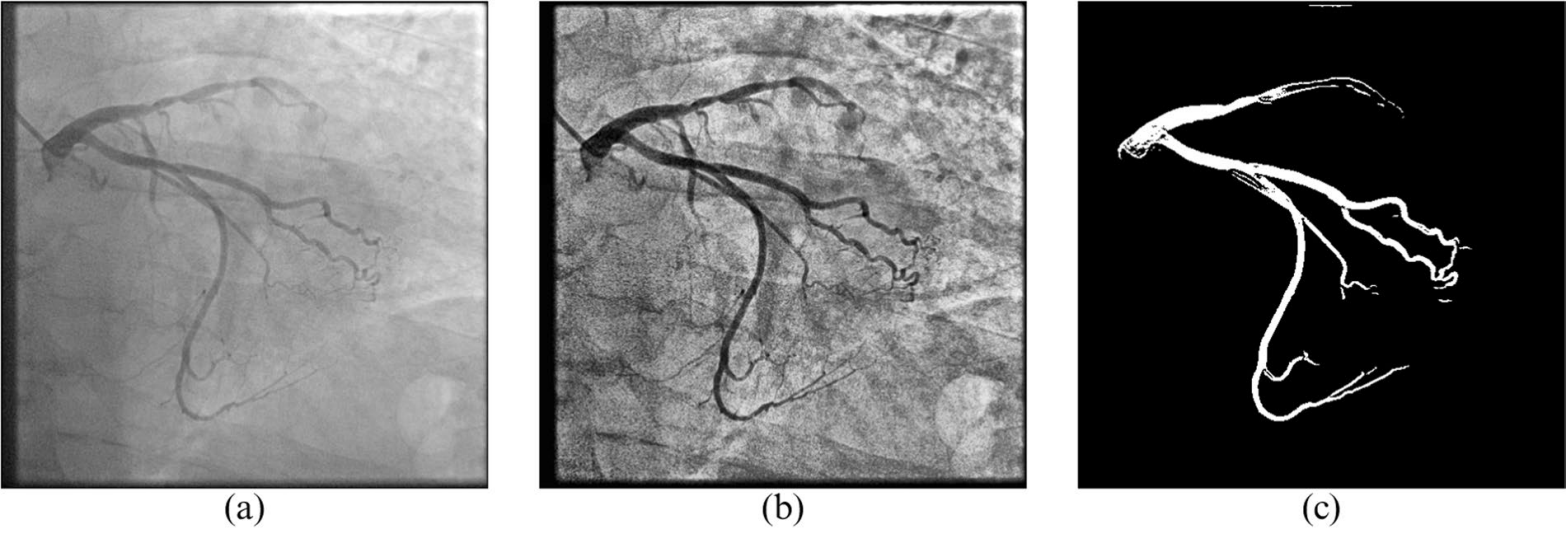
Images used to train four YOLOv8 models: (**a**) **original** X-ray image, (**b**) contrast- and sharpness-**enhanced** image, (**c**) predicted **binary** segmentation mask.
- Contrast- and sharpness-**enhanced** XCA images (Fig. 4b).
- **Binary** masks of XCA images that were previously generated by our U-Net model (Fig. 4c).

To show the effect of image pre-processing on model performance, YOLOv8 was trained with three different types of images:

- **Original** XCA images (Fig. 4a).

To evaluate the performance of YOLOv8, we calculate Precision, Recall, and Dice Scores for all stenoses, vessels, and for each SYNTAX class for the 300 test-set images. These results can be obtained from Table 2, where the “Class” column represents the specific target, the “Images” column represents the number of images in the test set, which is equal to 300, and the “Instances” column indicates the total number of class instances in the test set. Original, Enhanced, and Binary represent the performance of the three models, which were trained with images shown in Fig. 4. The “stenosis” and “vessels” rows in this table represent the average scores for the stenosis detection and vessel segmentation objectives, while subsequent rows represent the segmentation metrics for specific vessel classes, i.e. RCA proximal, mid, and distal are presented as classes 1,2, and 3 respectively, as given in the SYNTAX definition. This table also provides insight into the distribution of LAD, LCX, and RCA views in the dataset. There are exactly 100 RCA images, which are represented by 100 instances of all 3 major RCA segments, and 195 LCA images, represented by 195 Left Main Coronary Artery (LMCA - Class ‘5’ in the Table 2) instances. There are slightly more LCA caudal views with 119 instances of SYNTAX ‘13’ (LCX distal), than LCA cranial with 83 instances of SYNTAX ‘7’ (LAD mid). While SYNTAX Class ‘7’ usually can not be as clearly identified in LAO and RAO caudal views, focusing on LCX, most of the time SYNTAX ‘6’ is annotated and analyzed in these views as well as in PA and RAO cranial views, which focus on the LAD, and this is why the number of occurrences of SYNTAX ‘7’ is significantly lower than the number of occurrences of SYNTAX ‘6’. Also, the visibility and diagnostic importance of side branches, such as ‘9’, ‘10’, and all classes that contain letters in their index, is small, so their number of instances is significantly smaller than that of larger vessels.

**Table 2:** Performance of YOLOv8x on different data configurations.

Class	Images	Instances	Original			Enhanced			Binary		
			Prec	Rec	Dice	Prec	Rec	Dice	Prec	Rec	Dice
stenosis	300	386	0.33	**0.45**	0.38	**0.36**	**0.45**	**0.40**	0.33	0.35	0.34
vessels	300	1672	**0.53**	0.46	**0.49**	0.46	**0.48**	0.47	0.45	0.37	0.41
1	300	100	0.61	0.83	0.70	**0.67**	**0.84**	**0.74**	0.60	0.78	0.68
2	300	100	0.56	0.79	0.66	**0.65**	0.79	**0.72**	0.56	**0.82**	0.67
3	300	100	**0.80**	0.71	**0.75**	0.62	0.78	0.69	0.60	**0.81**	0.69
4	300	92	**0.71**	0.66	**0.69**	0.65	**0.67**	0.66	0.62	0.54	0.58
5	300	195	0.81	**0.86**	**0.84**	**0.88**	0.79	**0.84**	0.74	0.78	0.76
6	300	188	0.66	**0.82**	0.73	**0.73**	0.77	**0.75**	0.59	0.64	0.61
7	300	83	0.50	0.76	0.60	**0.52**	**0.77**	**0.62**	**0.52**	0.64	0.57
8	300	80	0.50	**0.65**	0.57	0.56	0.62	**0.59**	**0.60**	0.33	0.43
9	300	66	0.20	0.33	0.25	0.34	**0.39**	**0.36**	**0.35**	0.36	**0.36**
9a	300	35	**0.43**	0.38	**0.41**	0.40	**0.40**	0.40	0.23	0.14	0.18
10	300	14	1.00	0.00	0.00	0.36	**0.21**	0.27	**0.98**	**0.21**	**0.35**
11	300	119	0.66	**0.88**	0.76	**0.71**	0.87	**0.78**	0.70	0.71	0.70
12	300	15	0.08	0.20	0.11	**0.18**	**0.27**	**0.21**	0.11	0.07	0.08
12a	300	23	**0.12**	**0.26**	**0.16**	0.04	0.12	0.06	0.08	0.17	0.11
13	300	110	0.49	0.58	0.53	**0.51**	**0.64**	**0.56**	0.39	0.44	0.41
14	300	67	0.57	0.21	0.31	**0.60**	**0.27**	**0.37**	0.49	0.23	0.31
14a	300	27	**0.50**	0.04	0.07	0.36	0.06	0.11	**0.50**	**0.15**	**0.23**
15	300	11	**0.10**	**0.09**	**0.10**	0.00	0.00	0.00	0.00	0.00	0.00
16	300	88	0.64	**0.70**	0.67	**0.68**	0.68	**0.68**	0.58	0.69	0.63
16a	300	24	**0.54**	0.42	**0.47**	0.31	**0.46**	0.37	0.35	0.12	0.18
16b	300	24	**0.47**	**0.44**	**0.45**	0.33	0.33	0.33	0.22	0.12	0.16
16c	300	25	1.00	0.00	0.00	0.34	**0.23**	**0.27**	**0.59**	0.08	0.14
12b	300	49	**0.47**	**0.31**	**0.37**	0.30	0.22	0.25	0.29	0.14	0.19
14b	300	37	0.18	0.14	0.15	**0.20**	**0.24**	**0.22**	0.00	0.00	0.00

Table 2 shows that the ‘Enhanced’ stenosis detection model showed the best performance for both Precision and Recall (0.36 and 0.45, respectively), resulting in the highest Dice score of 0.40. For the vessel segmentation task, the ‘Original’ model shows the highest Precision (0.53), and the ‘Enhanced’ model shows the highest Recall (0.48). In terms of Dice score, the ‘Original’ model showed a Dice score of 0.49, slightly outperforming the contrast-enhanced model.

From the results obtained from this table, it is evident that contrast enhancement can reduce the number of false positive responses of a model for the stenosis detection task while keeping the proportion of false negatives the same in both objectives.

**Fig. 5:**
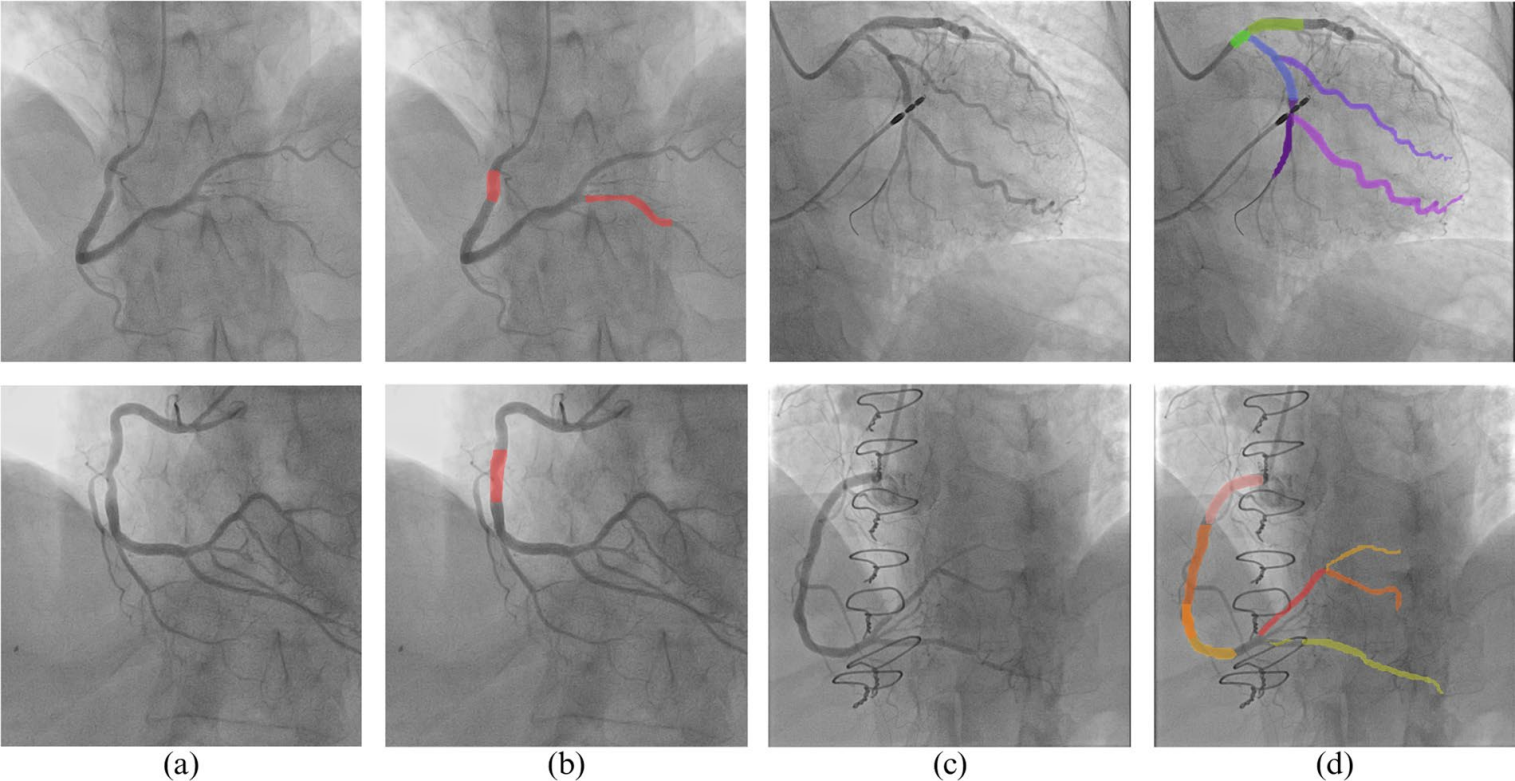
YOLOv8 results on out-of-distribution images: (**a**) and (**c**) original images; (**b**) stenosis detection result using the proposed method; (**d**) coronary region classification results using the proposed method.

The quantitative results of stenosis detection and vessel region classification can be obtained from Fig. 5. The results indicate that the model can accurately segment major stenotic regions even in the presence of foreign objects such as sutures and a catheter, while also correctly identifying vessel regions. However, it struggles to segment less significant and less present vessels, such as obtuse margins and diagonals, and struggles to identify the starting and ending edges of the vessels.

## Usage Notes

The dataset is available to download at 10.5281/zenodo.10390295. The dataset can be utilized to train and evaluate machine learning models for lesion detection and coronary vessel classification, synthetic data generation, semantic segmentation of vessels, X-ray image denoising, and many other potential applications.

Image processing techniques, such as the contrast enhancement and the binary segmentation model, as well as annotation conversion tools used in this article, can be found in the repository under the Code Availability section. All related project data are freely available under a CC0 license.

## Data Availability

The necessary scripts for this study are available in the GitHub repository (https://github.com/cmctec/ARCADE). This repository contains scripts to convert COCO annotation files into 2D segmentation masks and YOLOv8 labels format (and back), contrast enhancement scripts, scripts for evaluating vessel and stenosis segmentation models, and code for training and post-processing binary segmentation results.
